# Uropathogenic *Escherichia coli* population structure and antimicrobial susceptibility in Norfolk, UK

**DOI:** 10.1093/jac/dkad201

**Published:** 2023-06-26

**Authors:** Cailean Carter, Alexandra Hutchison, Steven Rudder, Elizabeth Trotter, Emma V Waters, Ngozi Elumogo, Gemma C Langridge

**Affiliations:** Microbes in the Food Chain, Quadram Institute, Norwich NR4 7UQ, UK; Medical School, University of East Anglia, Norwich NR4 7TJ, UK; Norfolk and Norwich University Hospital Microbiology Department, Norwich Research Park Innovation Centre, Norwich NR4 7GJ, UK; Microbes in the Food Chain, Quadram Institute, Norwich NR4 7UQ, UK; Medical School, University of East Anglia, Norwich NR4 7TJ, UK; Norfolk and Norwich University Hospital Microbiology Department, Norwich Research Park Innovation Centre, Norwich NR4 7GJ, UK; Microbes in the Food Chain, Quadram Institute, Norwich NR4 7UQ, UK; Norfolk and Norwich University Hospital Microbiology Department, Norwich Research Park Innovation Centre, Norwich NR4 7GJ, UK; Microbes in the Food Chain, Quadram Institute, Norwich NR4 7UQ, UK

## Abstract

**Background:**

Urinary tract infections (UTIs) are a frequent cause for visits to primary care providers. In alignment globally, uropathogenic *Escherichia coli* (UPEC) are the main aetiological agent for UTIs in Norfolk and are increasingly difficult to treat due to multi-drug resistance.

**Objectives:**

We set out to identify which clonal groups and resistance genes are disseminating in the community and hospitals in Norfolk, the first study of its kind for UPEC in this region.

**Methods:**

We collected 199 clinical *E. coli* isolates causing UTIs in the community and hospital from the Clinical Microbiology laboratory at Norfolk and Norwich University Hospital between August 2021 and January 2022. These were whole-genome sequenced using the Illumina and MinION platforms for *in silico* MLST and antibiotic resistance determinant detection.

**Results:**

The isolates were composed of 70 STs; 8 lineages represented 56.7% of this population: ST73, ST12, ST69, ST131, ST404, ST95, ST127 and ST1193. Importantly, primary UTI screening deemed 6.5% of isolates to be multidrug resistant (MDR), with high rates of resistance to ampicillin (52.1%) and trimethoprim (36.2%) in hospitals. Of concern is the probable clonal expansion of MDR groups ST131 and ST1193 in hospitals and community settings with chromosomally encoded *bla*_CTX-M-15_, *bla*_OXA-1_ and *aac(6′)-Ib-cr5*.

**Conclusions:**

The burden of reported UTIs in Norfolk is largely caused by non-MDR isolates and mirrors similar UPEC studies nationally and internationally. Continually monitoring samples with consideration of sources will help reduce burden of disease.

## Introduction

Urinary tract infections (UTIs) involving uropathogenic *Escherichia coli* (UPEC) are a significant contributor for both visits to primary care providers and antibiotic prescribing in the UK. Besides monitoring antibiotic prescribing practices and antimicrobial resistance testing results for UTIs, England does not have comprehensive UTI surveillance to monitor national or regional UPEC populations.^1^ Therefore, independent regional studies report emerging MDR clonal groups using sequence typing. One example is the emergence of ST131 in the early 2000s, arguably the most successful group in its frequency and acquisition of MDR determinants.^2^ The globally emerging ST1193 (first recorded in 2012) has gained interest internationally for acquisition of fluoroquinolone resistance from ST10 and subsequent clonal expansion.^3–5^

In this work, we investigated the UPEC population circulating in Norfolk, a rural county in East Anglia with a population of 916 200 people, in 2021/22.^6^ In this region, 67.5% of reported UTIs are from women, of all ages.^7^ However, the remaining third are reported by men, mostly between the ages of 66 and 85 years. This is significant as elderly men have been strongly associated with uncomplicated UTIs caused by MDR *E. coli* carrying ESBLs.^8,9^ The expansion of MDR clonal groups limits therapeutic options and can mean deviating from cost-effective options. Such a trend may have contributed to the expected cost of £860 161 in 2021 for 88 459 antibiotic prescriptions for uncomplicated UTIs under NHS Norfolk and Waveney.^10^ Thus, we sought to better understand the landscape of UPECs being reported at community and hospital facilities by WGS and antibiotic resistance phenotyping.

## Materials and methods

### Collection of E. coli isolates from UTI cases

A total of 199 clinical *E. coli* isolates from UTI patients were collected from the Norfolk and Norwich University Hospital (NNUH) Clinical Microbiology laboratory between August 2021 and January 2022, irrespective of phenotype and patient attributes (Table S1, available as Supplementary data at *JAC* Online). Isolates were collected equally from GP practices (defined as community) and tertiary care facilities (hospitals) in Norfolk. Only samples that were positive for infection (> 35 WBC/μL and/or ≥9000 bacteria/μL) determined by the Sysmex UF 5000 Automated Microscopy system and 10^5^ cfu/L on orientation (ORI) agar chromogenic plates were included. Repeat urine specimens were included during the collection period and records of repeat samples were kept. Four patients provided repeat samples: three patients provided two samples and one patient provided three samples. For each sample, a single purple colony on an ORI plate, confirmed to be *E. coli* and isolated from mid-stream and catheter specimens of urine was selected, streaked onto LB agar, and incubated overnight at 37°C. Subsequently, 1 mL of pre-warmed LB broth was added to each sample and incubated at 37°C overnight to prepare stocks in 20% glycerol that were stored at −80°C.

### Phenotypic resistance

Phenotypic resistance metadata was provided by NNUH. A first-line urine profile was performed with ampicillin, cefalexin, cefpodoxime, ciprofloxacin, co-amoxiclav, gentamicin, nitrofurantoin and trimethoprim using Oxoid discs following the 2021 EUCAST (v 9.0) guidelines.^11^ Cefpodoxime was used for screening AmpC and/or ESBL production but is not part of the NHS formulary and is therefore not reported in this study.^12^ When the criteria for MDR were passed (resistance identified to nitrofurantoin and trimethoprim, ampicillin/co-amoxiclav and cefalexin, or cefpodoxime), isolates were put forward for further antibiotic screening on the VITEK 2 system (bioMérieux) with N351 Gram-negative susceptibility cards. The susceptibility to (piv)mecillinam and aztreonam was determined by disc diffusion for MDR isolates. Visualization of phenotypic resistance was generated with Adobe Illustrator.

### WGS

Fire Monkey DNA Extractions Kits (RevoluGen) were used for DNA extraction, with the first 98 being performed manually using spin columns, and the remaining 101 being processed using Fire Monkey filters on a 96-well plate and positive air pressure on the Resolvex A200 robotic platform (Tecan). Manual DNA extracts were quantified using Qubit with a broad-range assay kit (Thermo Scientific); automated DNA extracts were quantified using Quant-IT broad range (Thermo Scientific) with a plate reader. Illumina sequencing was performed on the NextSeq as described by Parker and colleagues.^13^ Quality control of short reads was performed as described in Method S1 and summarized in Table S2.

### In silico analysis

Most *in silico* analyses were performed on the Quadram Institute cloud-based Galaxy server (release 19.05).^14^ Command-line tools were run on Ubuntu 20.04 LTS on Windows Subsystem for Linux 2. Python3 (v3.8.2) was used for data handling on Jupyter Notebooks with the pandas (v1.1.3) and NumPy (v1.18.3) packages. Fisher’s exact test and the chi-squared test were performed using SciPy (v1.4.1). Unless otherwise stated, default parameters were used. Code used for this study can be found at https://github.com/CaileanCarter/UPEC_pop_study_Norfolk.

### Draft whole-genome assemblies

Short-read sequences were assembled with Shovill (v1.1.0)^15^ with down-sampling to an average depth of 100×, estimated genome size of 5 Mb, minimum contig length of 500 bp, minimum coverage of 2, and using the SPAdes assembler. Assembly completeness was inspected using BUSCO (v3.0.2)^16^ on the FASTA contig files.

### In silico typing

Sequence typing was performed using MLST (v2.16.1).^17,18^ Phylogrouping was performed using EzClermont (v0.6.3).^19^  *fimH* typing of ST131 isolates was performed by making a BLAST database from a *fimH* allele database.^20^ Isolates of ST131 were aligned against the *fimH* database using BLASTn with default parameters. Results were filtered for 100% identity and 100% positive matches (ppos). Assigning ST131 subclades was performed as described by Stoesser and colleagues.^21^ Plasmid typing by MLST was performed *in silico* using PlasmidFinder (v2.0.1) with the PlasmidFinder database (accessed 29 September 2022) and matches were filtered for ≥95% identity and ≥95% coverage.^22^ Size of plasmid contigs were calculated using the *Compute sequence length* tool (Galaxy v1.0.1) in Galaxy.^23^ Plasmid alignments and visualization were performed as described in Method S2.

### Core-genome alignment, phylogeny and clustering

To investigate the relationships in our dataset from the perspective of the core genome, a core-genome alignment of short reads against *E. coli* UTI89 (NC_007946.1; GCF_000013265.1) was made using Snippy (Galaxy v4.4.3 + galaxy2).^24^ On average, 84% ± 0.055% of the reference genome was aligned with sequencing reads. Snippy-core was used to combine the Snippy output into a single core SNP alignment for constructing a maximum-likelihood (ML) phylogenomic tree with RAxML (v8.2.4).^25^ Visualization and annotation was done in R (v4.2.1) using RStudio (v2202-07-22 for Windows). The R package APE (v5.6-2) was used to load the Newick file of the best-scoring ML tree from RAxML.^26^ Plotting of the tree and metadata was done with ggtree (3.4.2) and ggplot2 (v3.3.6).^27–30^ Clustering of isolates was supported by the FastBAPS Bayesian hierarchical clustering algorithm using a core-genome alignment and ML tree, and the ‘optimise.symmetric’ prior.^31^ Robustness of FastBAPS clustering was confirmed with a heatmap as described in the FastBAPS documentation. Any isolates not automatically assigned a phylogroup (*n* = 4) or ST (*n* = 13) were assigned to a plausible group by inference from the neighbouring isolates within the same subclade. SNP distances were calculated using snp-dists (Galaxy v0.6.3 + 0) with the core SNP alignment as input.^32^ A cut-off of 17 SNPs was used to define transmission events (namely putative clusters) between patients as described by Ludden and colleagues.^33^

### Identification of AMR determinants and association with phenotypic resistance

Draft assemblies were annotated and translated into amino acid sequences using Prokka (v1.14.5).^34^ Annotation of AMR determinants was performed by AMRFinderPlus (v3.10.40) with inputs from Prokka and FASTAs of draft assemblies.^35^ As we could only identify a nitrofurantoin resistance determinant for one of two nitrofurantoin-resistant isolates, a database of nitrofurantoin resistance determinants in *E. coli*, NITREc, was used.^36^ The UPEC amino acid sequences were aligned against this database using BLASTp (v2.10.1) and filtered for 100% identical matches and 100% positive-scoring matches. Association of resistance determinants with phenotypic resistance was calculated by Fisher’s exact test and corrected for false discovery rate using the Python statistics package statsmodels’ (v0.12.2) *fdrcorrection* method. To improve confidence of association, a chi-squared (χ^2^) test with Yates’ correction was used, and genes were filtered by statistical significance agreed by both tests. Strength of association, Cramér’s phi (φ_c_), was calculated from the χ^2^ with the formula $\varphi_{c}=\sqrt{\chi^{2}÷num.\;isolates}$. Relationship between source and antibiotic resistance phenotype was calculated by χ^2^ with Yates’ correction. Isolates found to have the *bla*_OXA-1_, *bla*_CTX-M-1_, *bla*_CTX-M-15_, *bla*_SHV-1_, *bla*_DHA-1_ or *aac(6′)-Ib-cr5* genes were long-read sequenced via Oxford Nanopore Technologies (ONT) to confirm genomic location. Contigs where the *rep* gene was identified by PlasmidFinder were cross-referenced with results from AMRFinderPlus for matching contig accessions, therefore connecting resistance genes with a given plasmid type.

### Native-ligation Nanopore sequencing for AMR determinant location

Forty samples from this project were prepared with another eight in a single 48-plex MinION library using the Ligation Sequencing Kit (SQK-LSK109, ONT) in conjunction with the Native Barcoding Expansion 96 (EXP-NBD196, ONT). The library was loaded onto the flow cell according to the manufacturer’s instructions. Sequencing was performed on the MinION platform using R9.4 flow cells (FLO-MIN106, ONT) with a run time of 48 h. ONT MinKNOW software (v4.0.5) was used to collect raw sequencing data and ONT Guppy (v6.3.8) was used for local base-calling, de-multiplexing and barcode trimming of the raw data.

### Genome assembly via Nanopore for AMR determinant location

Draft assemblies from Nanopore reads were made with the Flye assembler (Galaxy v2.9.0) with the following inputs: mode was set to ONT regular reads pre-Guppy5, 5 Mb estimated genome size, five polishing iterations, and allowed to rescue short unassembled plasmids.^37^ Of the 40 samples long-read sequenced, 23 had sufficient reads for assembly. A polishing step was performed on the draft assemblies with the long reads using Medaka (Galaxy v1.4.3) to generate a consensus sequence; r941_min_high_g303 was used as the model.^38^ Two rounds of polishing using long reads with Racon (Galaxy v1.3.1.1) were performed; minimap2 (Galaxy v2.17.2) was used to provide the alignment of long reads to draft assemblies for polishing.^39,40^ Lastly, two rounds of polishing with short reads were performed with Pilon (Galaxy v1.20.1), also using minimap2 for alignment of short reads to draft assemblies.^41^ Each polishing stage was quality checked using CheckM (Galaxy v1.0.11) for assembly completeness, contamination and strain heterogeneity.^42^

## Results

### Composition of E. coli causing UTIs in Norfolk

**Figure 1.**
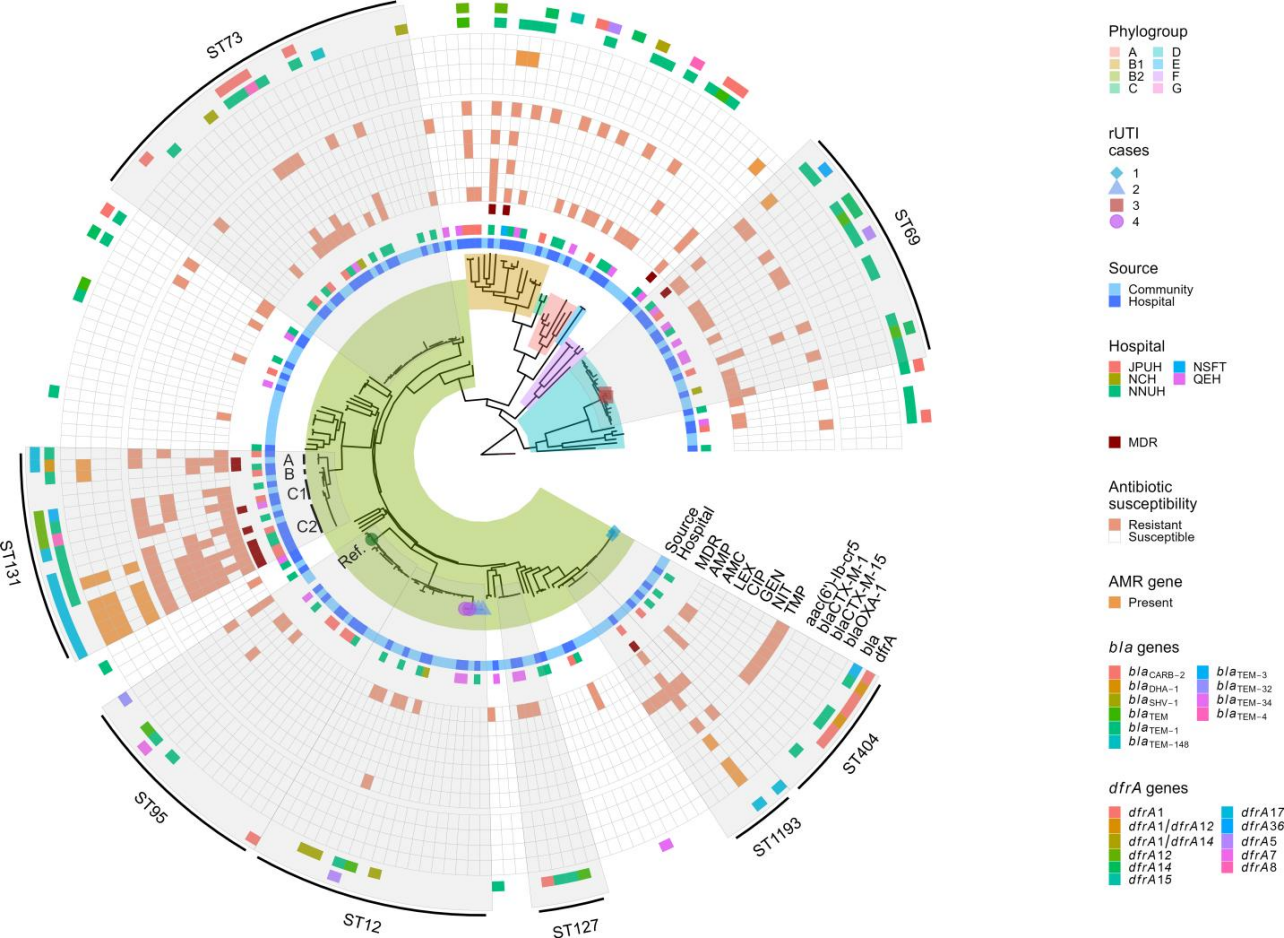
Phylogenomic tree of 199 *E. coli* isolates from UTI cases in Norfolk. Branch backgrounds are coloured by phylogroup. The reference genome, *E. coli UTI89*, is indicated by a dark green filled circle at the tip of the branch and labelled ‘Ref.’ at the tip. Four rUTI cases are numbered and colour/shape coded at the branch tips. ST131 subclades are labelled A, B, C1 and C2 at branch tips. Metadata visualized include: source and hospital of origin [James Paget University Hospital (JPUH), Norwich Community Hospital (NCH), NNUH, Norfolk & Suffolk Foundation Trust (NSFT) Waveny Ward and Queen Elizabeth Hospital (QEH) King’s Lynn]; isolates classified as MDR and with phenotypic antibiotic resistance to ampicillin (AMP), co-amoxiclav (AMC), cefalexin (LEX), ciprofloxacin (CIP), gentamicin (GEN), nitrofurantoin (NIT) and trimethoprim (TMP); presence of *aac(6′)-Ib-cr5*, *bla*_CTX-M-1_, *bla*_CTX-M-15_, *bla*_OXA-1_, other *bla* and all *dfrA* genes identified. Predominant STs are highlighted and labelled.

Using phylogroups as a broad typing method, and excluding repeat samples, *E. coli* from phylogroup B2 were responsible for most of the reported UTIs (71.1%) (Table 1). Other phylogroups each represented less than an eighth of the dataset with D (12.4%) and B1 (7.7%) being the second and third most common phylogroups, respectively. As isolates were found for all documented phylogroups of *E. coli*, this suggested that an evolutionarily diverse range of *E. coli* isolates were causing UTIs in Norfolk. Despite this diversity, a phylogenomic tree of the isolates collected revealed eight clonal groups comprising 56.7% of the dataset with 7/8 in phylogroup B2 (Figure 1). We identified 70 STs in our dataset, with ST73 as the most frequently isolated (Table 1). Of general concern were MDR groups ST131 and ST1193, which were in the top eight most frequently isolated clonal groups; however, the number of MDR isolates in each (8/17 and 1/6, respectively) was low. One isolate could not be assigned an ST by MLST due to mixed *E. coli* strains. The frequencies of ST12, ST69 and ST131 in this study were very similar and likely equally contributed to presentations of UTIs during time of collection.

**Figure 2.**
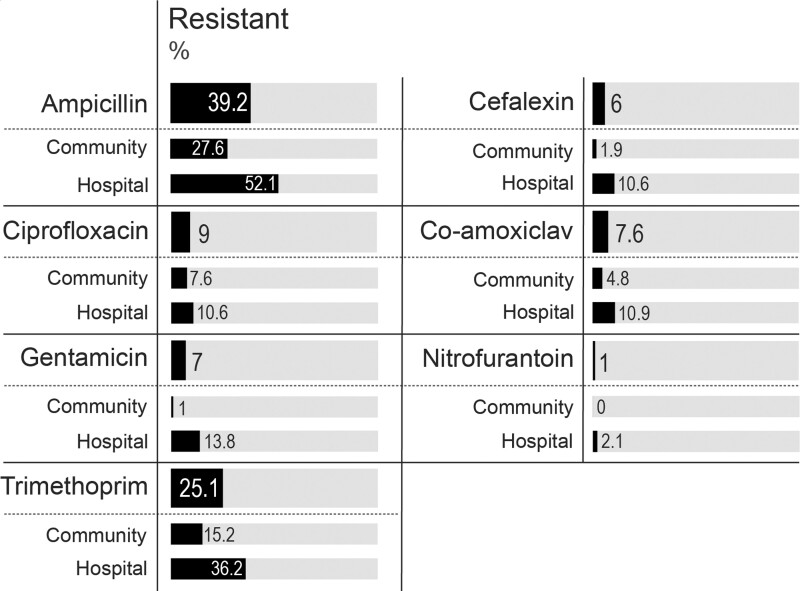
Antibiotic resistance rates. Percentage of isolates resistant to antibiotics used in first-line UTI treatment. Each antibiotic includes the total percentage (top) and the percentage of isolates found in community and hospital (below) resistant to that antibiotic. (Total *n* = 199, community *n* = 105, hospital *n* = 94).

**Table 1.** Phylogroups and predominant STs excluding repeat samples

Phylogroup	No. isolates (%)	ST	No. isolates (%)
B2	138 (71.1)	73	27 (13.9)
D	24 (12.4)	69	17 (8.8)
B1	15 (7.7)	131	17 (8.8)
A	6 (3.1)	12	16 (8.2)
F	6 (3.1)	95	11 (5.7)
C	2 (1.0)	404	10 (5.2)
E	2 (1.0)	1193	6 (3.1)
G	1 (0.5)	127	6 (3.1)
Total	194 (100)		110 (56.7)

We collected isolates from hospital (*n* = 94) and community (*n* = 105) settings to determine and compare the *E. coli* composition reported in each setting. There was a roughly equal distribution of sources across the phylogenomic tree, including most predominant clonal groups. This suggests frequent movement between settings, which is supported by the lack of clustering of isolates from a particular hospital (Figure 1; Table S3). However, ST131 was largely sourced from hospitals (12/17 isolates; 73%) and ST1193 from the community (5/6 isolates; 83%).

Four patients provided multiple samples for this study: three provided two samples and one patient provided three samples. In each case, the sequencing of successive samples revealed patients were either reinfected with the same isolate or were unable to clear the infection between samplings (Table S3). Interestingly, a branch deviated from the main ST12 cluster encapsulating five isolates from two separate recurrent (r)UTI cases (rUTI case #2 with three isolates, rUTI case #3 with two isolates), which has led to over-representation of ST12 in our dataset.

### Resistance rates for antibiotics used in UTI screening

Overall, there was mostly broad susceptibility (>90% of isolates susceptible to a given antibiotic) to antibiotics used in UTI screening (Figure 2). The highest resistance rate in hospital and community settings was observed for ampicillin, where just over half of hospital isolates (52.1%) and a quarter of community isolates (27.6%) were resistant to ampicillin. In contrast, the lowest resistance rate for hospital and community setting was observed for nitrofurantoin, the current first-line treatment for uncomplicated UTIs. No clustering of nitrofurantoin resistance was observed but could be found in predominant clonal groups, suggesting relatively recent acquisition of nitrofurantoin resistance determinants (Figure 1). Despite a high resistance rate for trimethoprim in hospital (36.2%) and community (15.2%) settings, not all of the predominant ST groups harboured trimethoprim resistance (ST95 and ST127); in those that did, the rate of resistance was highly variable (22%–76%). Indeed, large clusters of trimethoprim resistance were observed in ST404 and ST131, with other groups exhibiting sporadic trimethoprim resistance gain/loss events (Figure 1). The overall resistance rate for ciprofloxacin was 9% and could be largely attributed to two groups: ST1193 and a subclade of ST131 (namely C2). Treatment options for ST131 were severely limited, with two isolates resistant to all but one of the first-line antibiotics used in UTI screening (with susceptibility to nitrofurantoin).

We assessed the relationship between antibiotic resistance and source using a chi-squared test of independence, χ*^2^* (1, *n* = 199). This relationship was significant for ampicillin (χ*^2^* = 12.5, *P* = 0.0004), cefalexin (χ*^2^* = 6.67, *P* = 0.01), gentamicin (χ*^2^* = 12.57, *P* = 0.0004) and trimethoprim (χ*^2^* = 11.5, *P* = 0.0007), evidencing higher resistance rates for these antibiotics in hospital settings. However, this relationship was not significant for co-amoxiclav (χ*^2^* = 2.46, *P* = 0.117) and ciprofloxacin (χ*^2^* = 0.55, *P* = 0.46). The test could not be performed on nitrofurantoin due to an insufficient number of expectants.

### AMR determinants associated with phenotypic resistance

A curated database of known resistance mechanisms was used to identify resistance genes and point mutations associated with phenotypic antibiotic resistance within our dataset. Fisher’s exact test was used to determine the statistical significance of gene association with a given antibiotic resistance trait (*P* < 0.05) and the strength of the association was determined with φ_c_, which provides a numerical scale of weak (0) to strong (1) association.

Of the total 18 antibiotics screened in this study (including the additional screening for MDR isolates), 14 were β-lactams—emphasizing the importance of this family of antibiotics for UTI treatment. Thus, we assessed the association between ESBL-encoding genes with phenotypic resistance to β-lactams. Thirteen different β-lactamase determinants were detected across 80 β-lactamase-carrying isolates (Figure 1). Of these, *bla*_TEM-1_ was the most frequently identified β-lactamase (*n* = 52) and was strongly associated with ampicillin resistance (*P* < 0.005) (Table 2). However, isolates found with just the *bla*_TEM-1_ β-lactamase had inconsistent resistance profiles against penicillins and co-amoxiclav, suggestive of one or more undetermined resistance mechanisms. The most frequently identified ESBL determinant was *bla*_CTX-M-15_ (*n* = 11), which was strongly associated with cefalexin resistance (*P* < 0.005, φ_c_ 0.86). Indeed, 8/11 isolates carrying *bla*_CTX-M-15_ were resistant to all five cephalosporins tested (Table S1). This gene was predominantly found in MDR groups ST131 (*n* = 7) and ST1193 (*n* = 1), but also in ST85 (*n* = 2) and ST3177 (*n* = 1). Curiously, the *bla*_CTX-M-15_ found in the two ST58 isolates from their short-read assemblies were not found in the long-read assembly for either isolate (Table S4). Concerningly, *bla*_CTX-M-15_ frequently co-occurred with *bla*_OXA-1_, reducing susceptibility to β-lactam/inhibitor combinations, and *aac(6′)-Ib-cr5*, conferring resistance to aminoglycosides and fluoroquinolones.^43^ Through long-read assembly, we verified the location of *bla*_CTX-M-15_, *bla*_OXA-1_ and *aac(6′)-Ib-cr5* in ST131 (*n* = 4) and ST1193 (*n* = 1) to be chromosomally encoded (Table S4). In three of four ST131 isolates with this combination, the insertion was downstream of the methionine tRNA ligase *metG* and upstream of the glycine betaine uptake system cassette *yehWXYZ*. In the remaining ST131 isolate (GL163), the insertion was instead found 20 kb downstream of *yehWXYZ* and 848 bp upstream of the galactoside transport system cassette *mglABC*. In the ST1193 isolate, the insertion was found 2.4 kb from aspartate tRNA ligase *aspS* and 5.5 kb upstream of carboxy-S-adenosyl-L-methionine synthase *cmoA*. While also carrying an IncFIA plasmid, it was smaller in size compared with that found in ST131 (92.4 kb), differed in content, and harboured different aminoglycoside resistance determinants [*aph(3′′)-Ib* and *aph(6)-Id*] while still possessing *dfrA17* (Figure S1). In six of seven ST131 isolates with long-read sequencing data, an IncFIA plasmid (143 ± 5.6 kb) was identified, which either harboured *aadA5* and *dfrA17* (*n* = 4), *bla*_DHA-1_ and *dfrA17* (*n* = 1), or *bla*_TEM-1_ alone (*n* = 1) (Table S4). Two other ST131 isolates with *bla*_CTX-M-15_ were long-read sequenced: one isolate had the gene integrated into the chromosome 1.2 kb upstream of aspartate kinase III *lysC* and the other isolate carried an IncFIB plasmid (125 kb) harbouring the gene (Figure S1). A diversity of co-amoxiclav resistance determinants were identified but most were not statistically associated; this included *bla*_TEM-30_, *bla*_TEM-32_, *bla*_TEM-34_, *bla*_TEM-40_ and *bla*_TEM-148_ (Figure 1).^44^ Screening of carbapenem resistance was performed for a cohort of MDR isolates (*n* = 13) and carbapenem resistance was not detected in this study. Other β-lactamases detected in this study included *bla*_SHV-1_ (*n* = 5), *bla*_CARB-2_ (*n* = 1), *bla*_CTX-M-1_ (*n* = 1) and *bla*_DHA-1_ (*n* = 1). The *bla*_CTX-M-1_ gene was found on an IncFII plasmid (145 kb) from an isolate in ST69. Within ST12, the *bla*_SHV-1_ gene was found in one isolate to be inserted in the chromosome 21 bp downstream from the glycerol 3P regulon repressor *glpR* and carried on an IncFIB plasmid (78.9 kb) in another isolate (Figure S1).

**Table 2.** Association of antibiotic resistance genes and point mutations with phenotypic resistance

Gene	Phenotype	G+ P+	G− P+	G+ P−	G− P−	*P* value corrected	Cramér’s association	OR
*bla*_TEM-1_	AMP	48	30	4	117	7.17E−19	0.65	47
*bla*_CTX-M-15_	AMP	10	68	1	120	1.65E−03	0.26	18
*bla*_OXA-1_	AMP	6	72	0	121	1.04E−02	0.22	inf
*bla*_SHV-1_	AMP	5	73	0	121	2.52E−02	0.20	inf
*bla*_TEM-40_	AMC	2	13	0	184	1.65E−02	0.35	inf
*gyrA* D87N	CIP	15	3	0	181	2.05E−18	0.91	inf
*parC* S80I	CIP	15	3	2	179	1.80E−16	0.84	448
*gyrA* S83L	CIP	16	2	13	168	7.90E−13	0.66	103
*parC* E84V	CIP	7	11	0	181	1.23E−07	0.61	inf
*aac(6′)-Ib-cr5*	CIP	6	12	0	181	1.32E−06	0.56	inf
*parE* L416F	CIP	6	12	0	181	1.32E−06	0.56	inf
*parE* I529L	CIP	7	11	10	171	6.66E−04	0.34	11
*bla*_CTX-M-15_	LEX	10	2	1	186	5.73E−13	0.86	930
*bla*_OXA-1_	LEX	6	6	0	187	1.12E−07	0.70	inf
*aac(3)-IIe*	GEN	6	8	0	185	2.55E−07	0.64	inf
*aac(6′)-Ib-cr5*	GEN	5	9	1	184	2.12E−05	0.53	102
*aadA5*	GEN	6	8	3	182	1.37E−05	0.51	46
*aac(3)-IId*	GEN	6	8	3	182	1.37E−05	0.51	46
*aadA2*	GEN	4	10	5	180	5.48E−03	0.32	14
*nfsA* G131D	NIT	1	1	0	197	2.85E−02	0.71	inf
*dfrA1*	TMP	17	33	3	146	4.31E−08	0.46	25
*dfrA17*	TMP	12	38	0	149	1.60E−07	0.44	inf
*dfrA12*	TMP	7	43	0	149	1.93E−04	0.33	inf
*dfrA14*	TMP	6	44	1	148	4.01E−03	0.27	20
*dfrA5*	TMP	3	47	0	149	4.12E−02	0.21	inf

Genes are listed with phenotypes with statistically significant association. Phenotypes are resistance to ampicillin (AMP), co-amoxiclav (AMC), ciprofloxacin (CIP), cefalexin (LEX), gentamicin (GEN) and trimethoprim (TMP). The combinations of genotype (G) and phenotype (P) presence/absence (+/−) are listed that were used for input for Fisher’s exact test (*P* value corrected and OR). Cramér’s association was used to determine strength of associations. Inf denotes an infinite value resulting from division of 0.

Ciprofloxacin resistance in our dataset was mostly attributed to ST131 and ST1193. This resistance was strongly associated with point mutations in *gyrA* D87N, *gyrA* S83L and *parC* S80I, which co-occurred in the same 15 ciprofloxacin-resistant isolates (Table 2). In addition to point mutations in *gyrA* and *parC*, ciprofloxacin-resistant ST131 isolates also carried the *aac(6′)-Ib-cr5* gene, which was significantly associated with both ciprofloxacin and gentamicin resistance. Trimethoprim resistance was associated with one of several *dfrA* derivatives, with 6% of resistant isolates having two variations of *dfrA*. The most common was *dfrA1*, which was found in 34% of trimethoprim-resistant isolates. *dfrA17* was limited to MDR groups ST131 (*n* = 10) and ST1993 (*n* = 2) and was carried on IncFIA plasmids in both STs for all isolates long-read sequenced (*n* = 6) (Figure 1; Figure S1; Table S4). Nitrofurantoin resistance mechanisms were identified for one of two isolates using the above approach; therefore, a specialized database was used. The two nitrofurantoin-resistant isolates were found to have known resistance mechanisms through point mutations in *nfsA* and *nfsB* (exact point mutations were not specified by the database).

## Discussion

The composition of predominant STs causing UTIs in Norfolk is reflective of predominant groups causing UTIs and bacteraemia in similar reports across the UK, Europe and the USA but in varying proportions.^2,45–52^ Many of these STs are associated with animal sources (including food and wild and companion animals) as detailed by Riley^53^ and Vincent *et al*.,^54^ and can persist in the human gut.^55,56^ Largescale studies of UK ESBL-producing *E. coli* imply ST131 is largely associated with humans, faeces and sewage sources.^57,58^ Indeed, some UK rivers have been identified as contaminated with CTX-M-carrying ST131; thus, it is worth considering the contribution of Norfolk’s rivers and Broads as potential sources of ST131.^59^ Although there are reports of ST131 isolated from chickens, the subclade of ST131 (namely B/H22) attributed to this only accounted for 1 of 17 ST131 isolates collected in this study, suggesting poultry as an unlikely source of UTIs caused by ST131 in Norfolk.^57,58,60^ Given the recent emergence of ST1193, the potential sources of this group remain unclear.^3^ A recent phylogenomic analysis of 707 publicly available ST1193 sequences offer evidence to suggest companion dogs, urban-adapted birds, and wastewater as potential sources.^61^ Within ST131 and ST1193, all isolates carrying *bla*_CTX-M-15_, *bla*_OXA-1_ and *aac(6′)-Ib-cr5* that were long-read sequenced were found to reside on the chromosome; this has been reported globally with some suggesting this is indicative of another clonal expansion of ST131.^33,62–64^ We are in agreement with Ludden *et al.*,^33^ that given the endogenous source of ST131 (and plausibly ST1193), measures should be taken to limit endogenous infections. Interestingly, the broadly susceptible ST12 group has not previously appeared as a predominant causative agent of UTIs in the UK; however, it has been observed in Spain, France and the USA and causing bacteraemia in the UK.^47,52,65,66^ A limitation of this study is that we did not sample from surrounding environments, animals or foods to identify sources of UPEC isolates in Norfolk. Nonetheless, these results suggest Norfolk is no exception to the global movement of UPEC given its similarity to the rest of the UK and international populations.

Since 2014, trimethoprim resistance in UPEC in Norfolk hospitals marginally reduced from 40% to 36.2%, whereas community rates halved from 33% to 15.2%, possibly reflecting the change in antibiotic prescribing practices to nitrofurantoin as a first-line drug.^7^ It is interesting to see such a reduction in trimethoprim resistance over 8 years; a 12 month prospective study in Sweden found little change in trimethoprim resistance after reducing trimethoprim prescribing, suggesting that a time frame of years rather than months is needed to observe a reduction in trimethoprim resistance.^67^ Our reported resistance rates for trimethoprim closely match the reported rates by NHS Norfolk & Waveney CCG for 2021 Q3 to 2022 Q1.^68^ The overall resistance rate for nitrofurantoin is a third of the rate observed across the UK during the period of collection.^68^ Thus, current nitrofurantoin and trimethoprim resistance trends reported here and by the UK Health Security Agency suggest suitable efficacy in this region as first-line treatment for UTIs using current regional NICE guidelines. Ciprofloxacin has been used frequently as prophylaxis for prostate biopsies in Norfolk.^69^ However, the prevalence of ciprofloxacin resistance attributed to global MDR groups ST131 and ST1193, as seen here, raises concerns about the antibiotic’s efficacy as prophylaxis.^70^

In conclusion, the population of UPEC causing UTIs in Norfolk mirrors UPEC populations reported nationally and internationally. The ongoing evolution and presence of predominant clonal groups limits cost-effective treatment options for many UTI cases and continued monitoring can inform policy making to limit the burden of disease.

## Supplementary Material

dkad201_Supplementary_Data

## Supplementary data

Supplementary methods 1 and 2, Figure S1 and Tables S1 to S4 are available as Supplementary data at *JAC* Online.
