# Supplementary material for: Uropathogenic Escherichia coli population structure and antimicrobial susceptibility in Norfolk, UK
Source: J Antimicrob Chemother. 2023 Jun 26;78(8):2028–36. doi: 10.1093/jac/dkad201 (PMC10393884; doi:10.1093/jac/dkad201)
Supplement: dkad201_Supplementary_Data [file dkad201_supplementary_data.zip › Supplementary_Electronic_Materials (1).docx]

# Supplementary

**Supp. Method 1: Quality control of short-read data**

All short reads were quality controlled using fastp (v0.19.5) and visualised using MultiQC (v1.11) to check reads were adequate for assembly and genotyping.^71,72^ Read sets were deemed good quality with <5% duplication rate before filtering (low number of duplicate reads), GC content 50±5%, and >90% reads passing the quality and length filter. To assert an adequate number of reads were available for draft genome assembly, isolates were checked for short read coverage against the reference genome of *Escherichia coli* UTI89 (NC_007946.1; GCF_000013265.1) using samtools (Galaxy Version 2.0.1) depth for BAM files with Snippy.^73^ If coverage was <30X, those isolates were re-sequenced and the reads merged with the previous run. QC summary can be found in Table S2. Isolates were again confirmed as *E. coli* using RefSeq Masher Matches (v0.1.1) by asserting the top match was *E. coli.*^74^

**Supp. Method 2: Plasmid alignment**

For each ST and plasmid type combination, the isolate with the largest plasmid size per type was selected as reference. A BAM file for alignments of short reads against the reference plasmid were generated using Snippy and converted to SAM format with samtools Plasmid annotations were made using Prokka with outputs as GenBank. Lastly, plasmid alignments were viewed in BRIG (v0.95).^75^

**References**

71. Chen S, Zhou Y, Chen Y*, et al.* fastp: an ultra-fast all-in-one FASTQ preprocessor*.* *bioRxiv*, 2018: 274100.

72. Ewels P, Magnusson M, Lundin S*, et al.* MultiQC: summarize analysis results for multiple tools and samples in a single report*.* *Bioinformatics*, 2016; **32**: 3047-3048.

73. Li H, Handsaker B, Wysoker A*, et al.* The Sequence Alignment/Map format and SAMtools*.* *Bioinformatics*, 2009; **25**: 2078-2079.

74. Ondov BD, Treangen TJ, Melsted P*, et al.* Mash: fast genome and metagenome distance estimation using MinHash*.* *Genome Biology*, 2016; **17**: 132.

75. Alikhan N-F, Petty NK, Ben Zakour NL*, et al.* BLAST Ring Image Generator (BRIG): simple prokaryote genome comparisons*.* *BMC Genomics*, 2011; **12**: 402.

| **Table S2. QC summary statistics for short reads (n=199)** | | | | |  |
| --- | --- | --- | --- | --- | --- |
| **Stat** | **Mean** | **Q75** | **Q50** | **Q25** | |
| **N50** | 211 Kbp | 262 Kbp | 205 Kbp | 153 Kbp | |
| **No. contigs** | 150 | 113 | 86 | 68 | |
| **Largest contig** | 0.5 Mbp | 0.64 Mbp | 0.52 Mbp | 0.39 Mbp | |
|  |  |  |  |  | |
|  |  |  |  |  | |


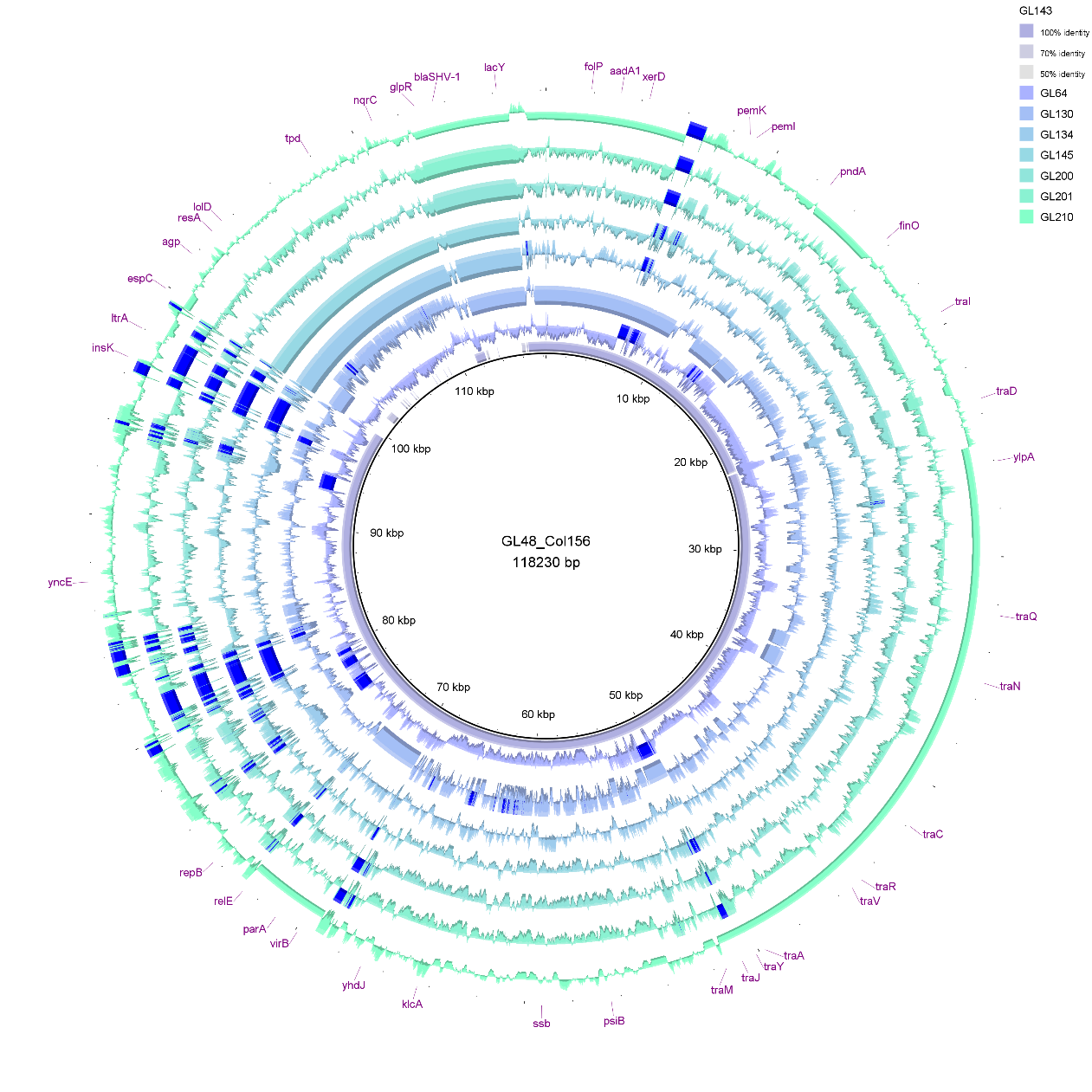

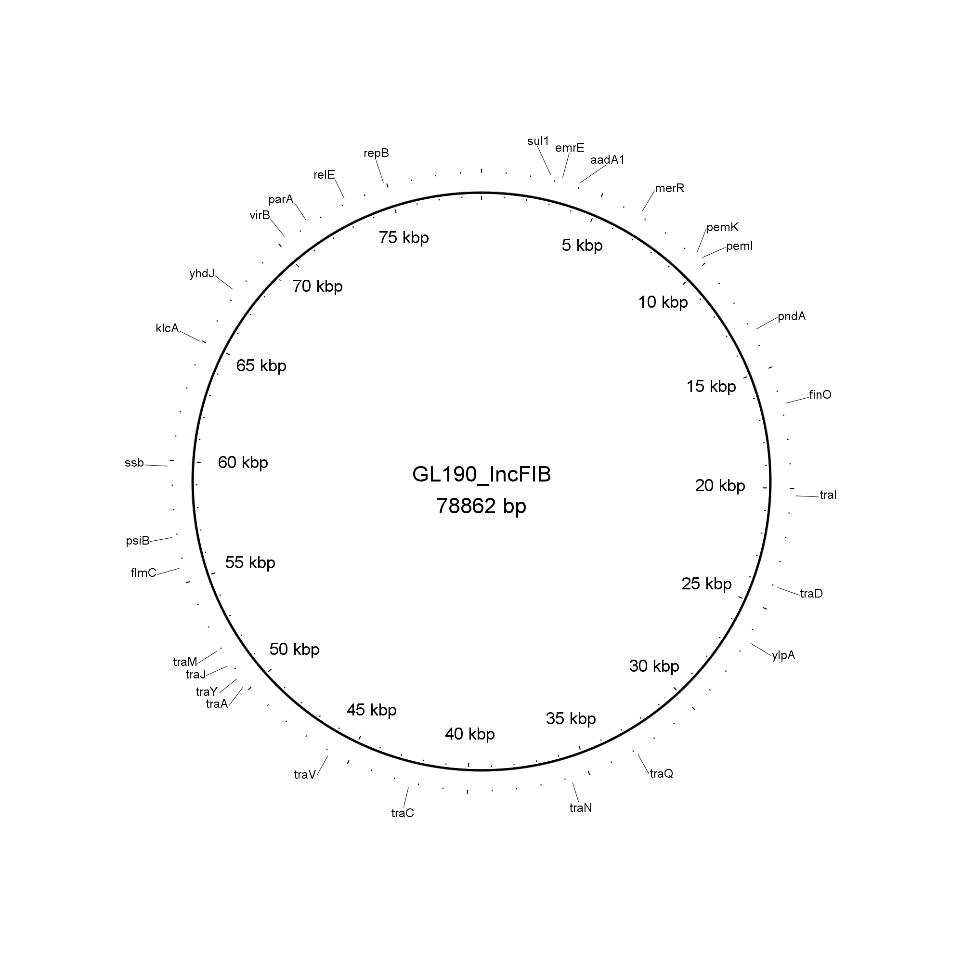


**ST12**


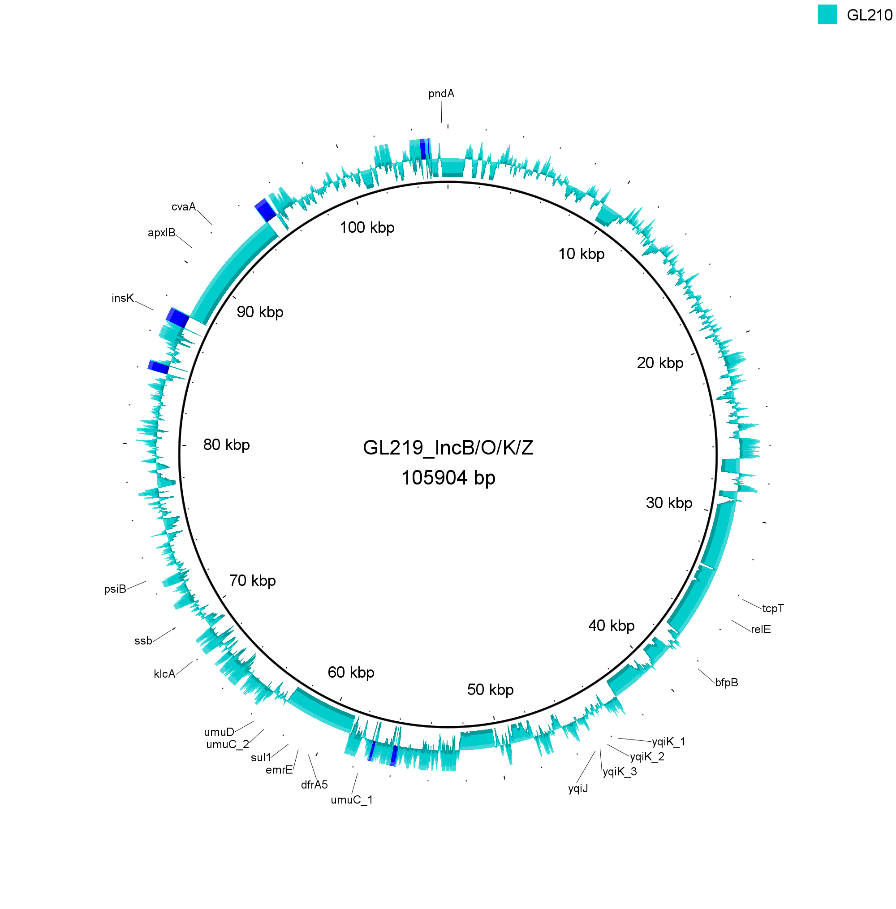

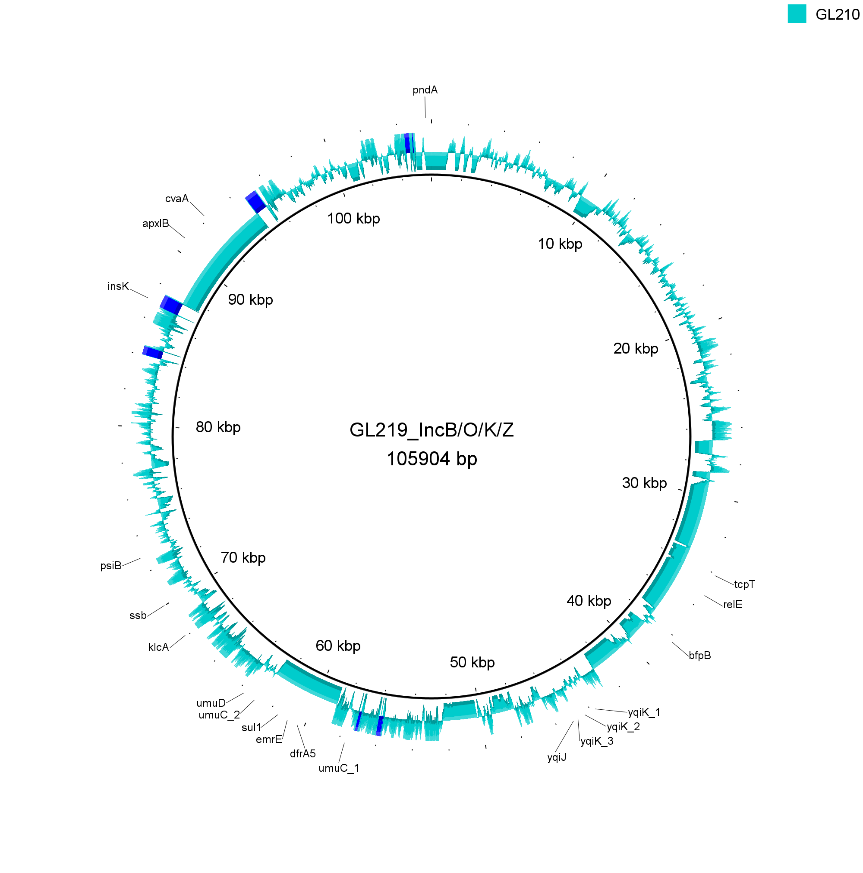


**ST69**


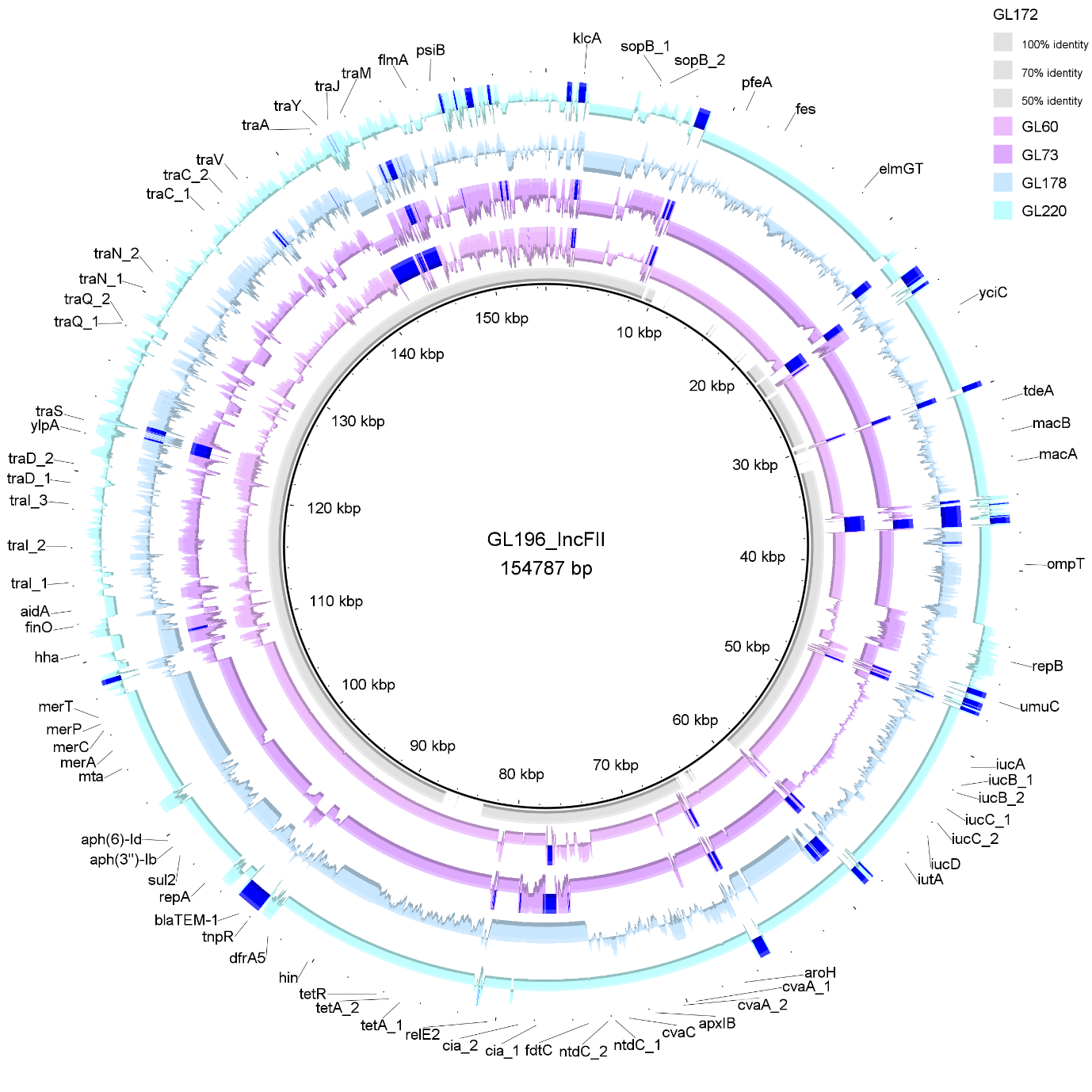


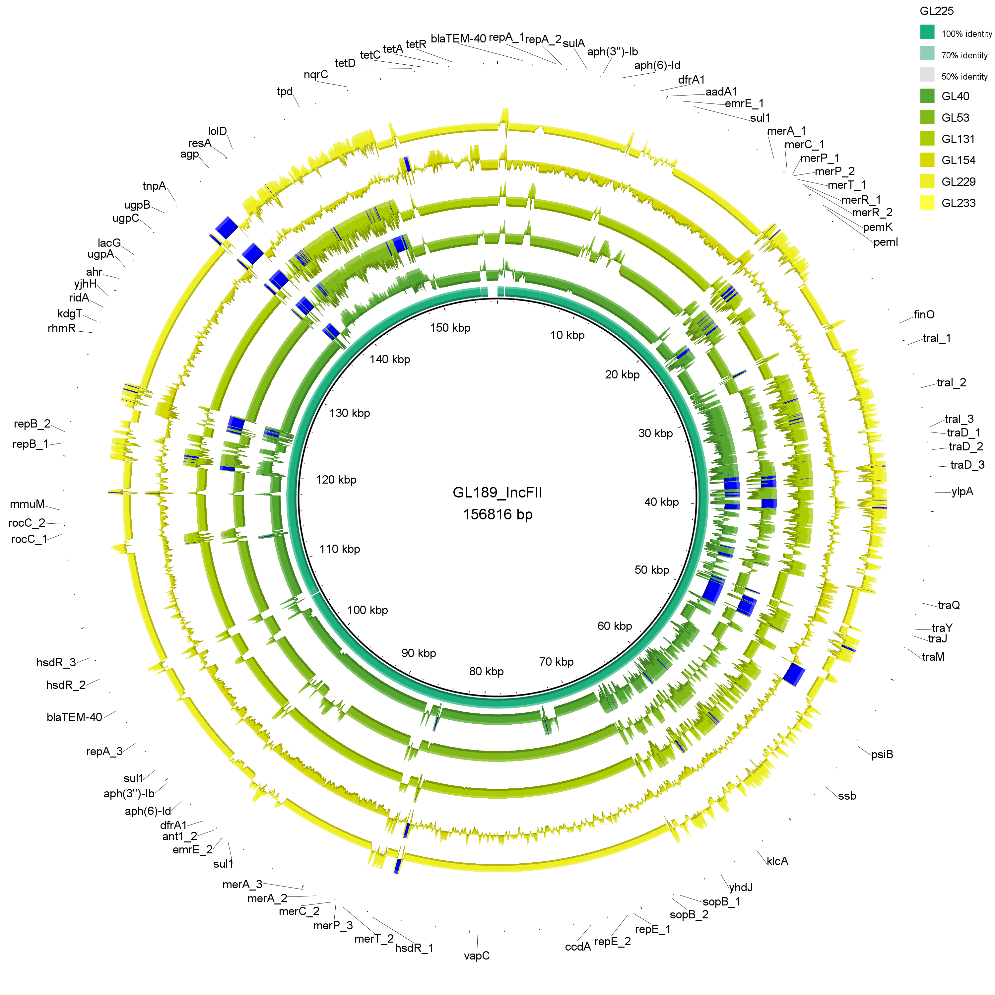

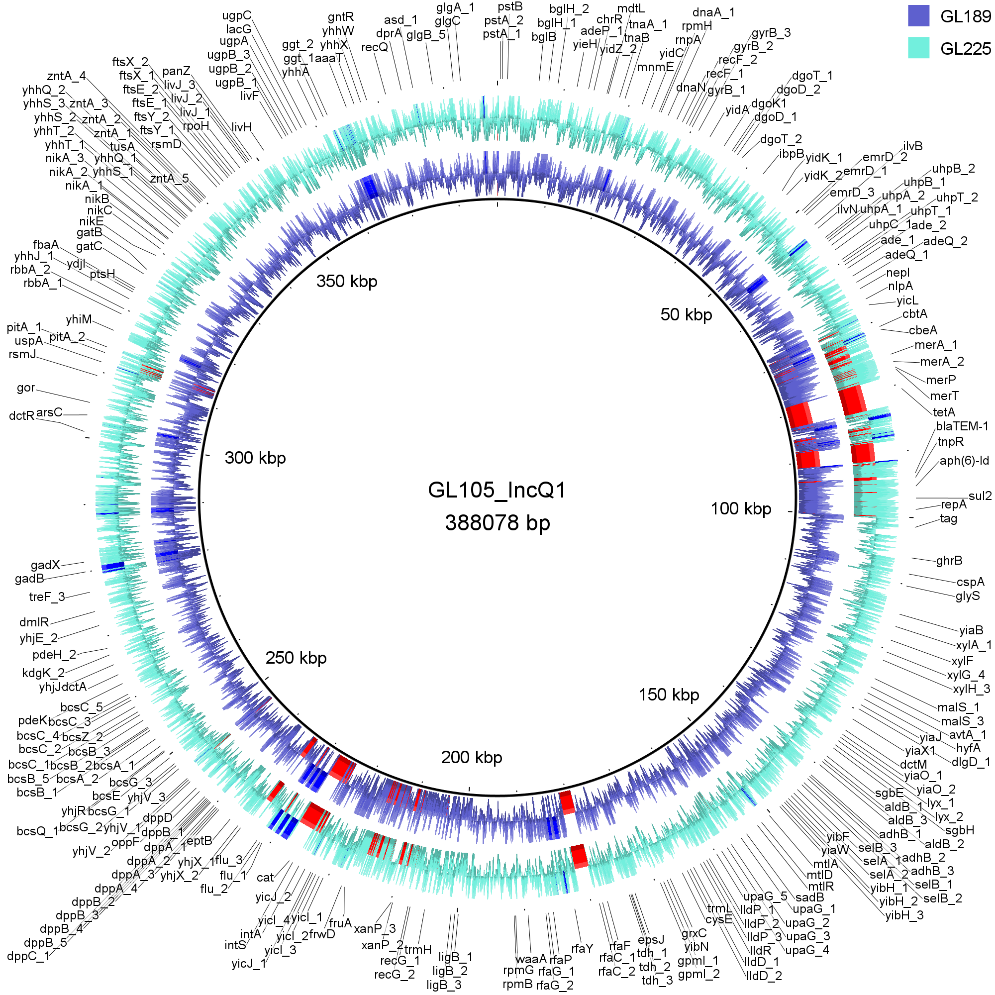


**ST73**

**ST79**


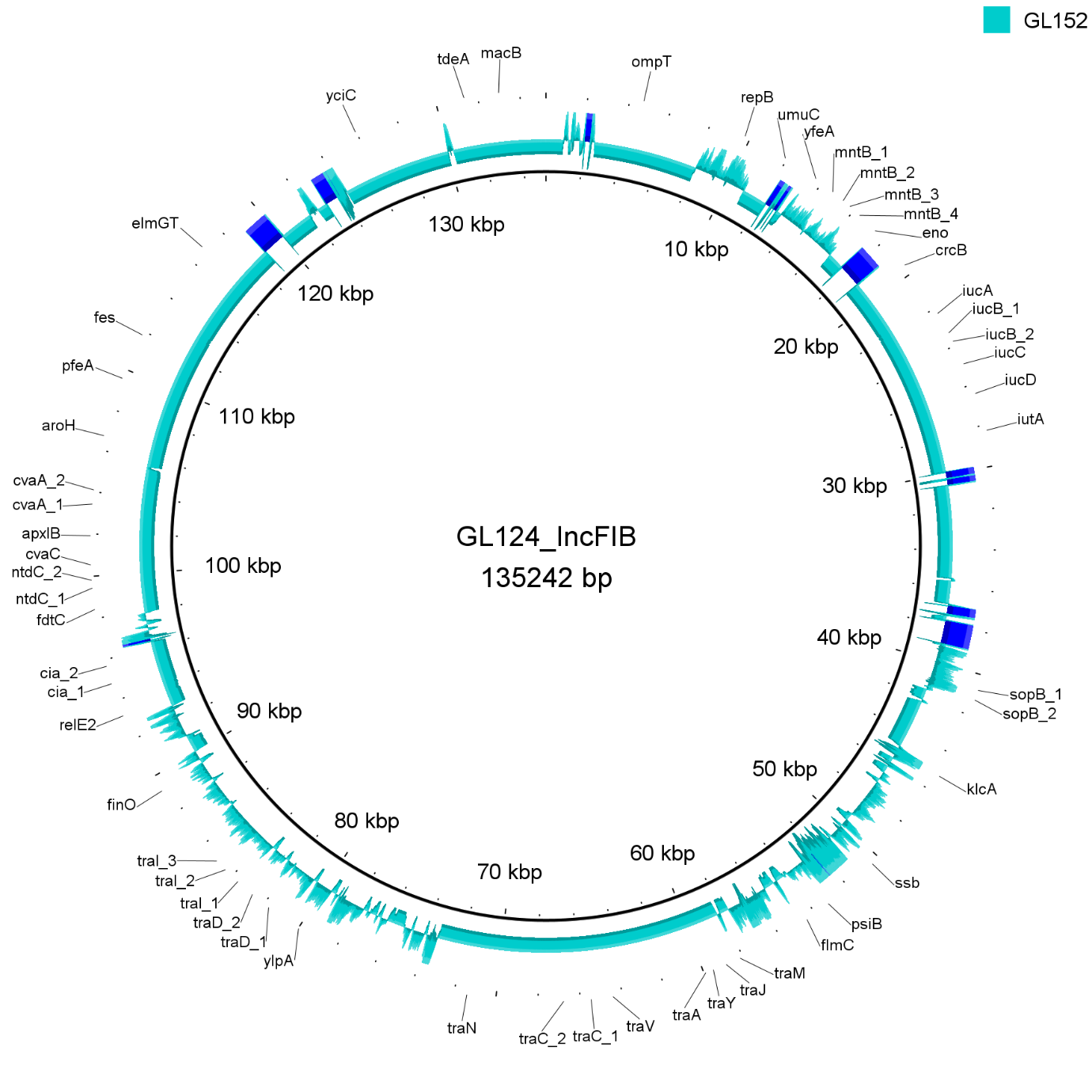


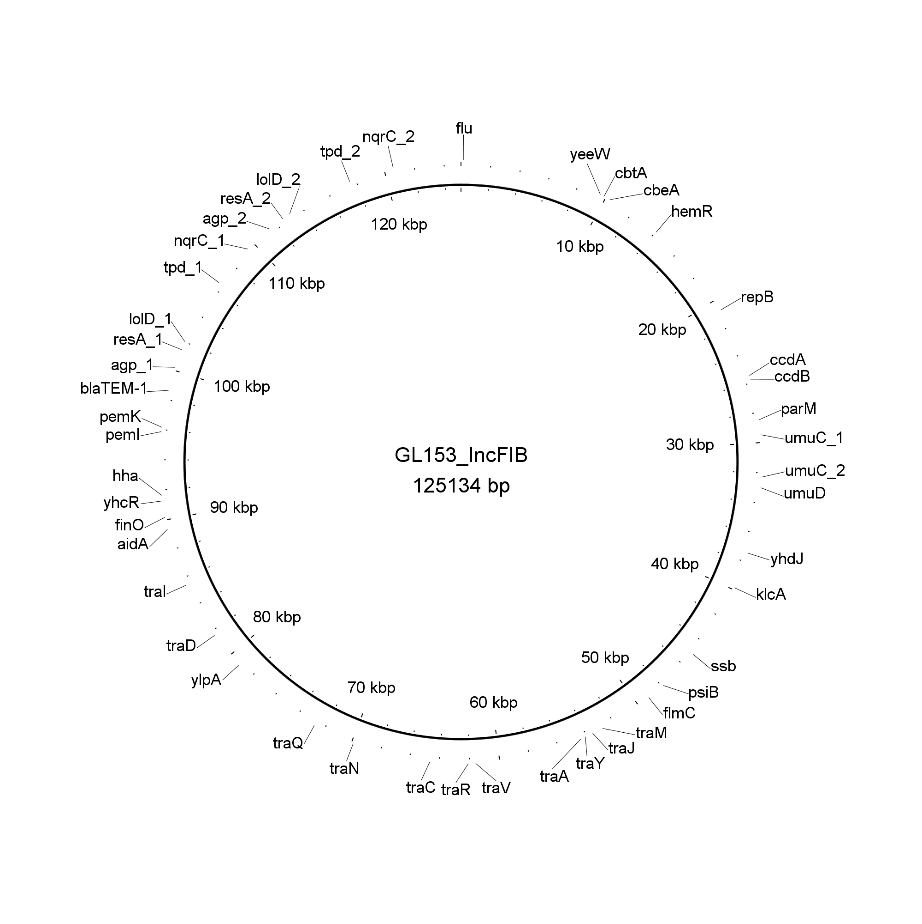

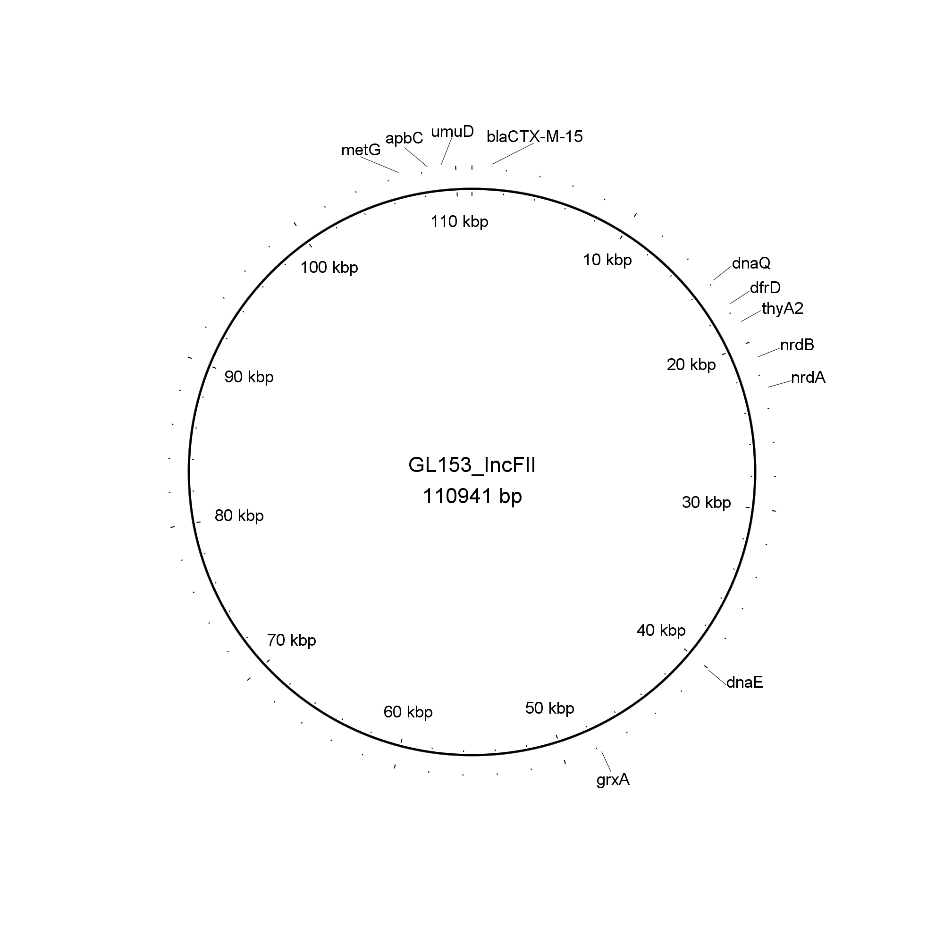


**ST131**


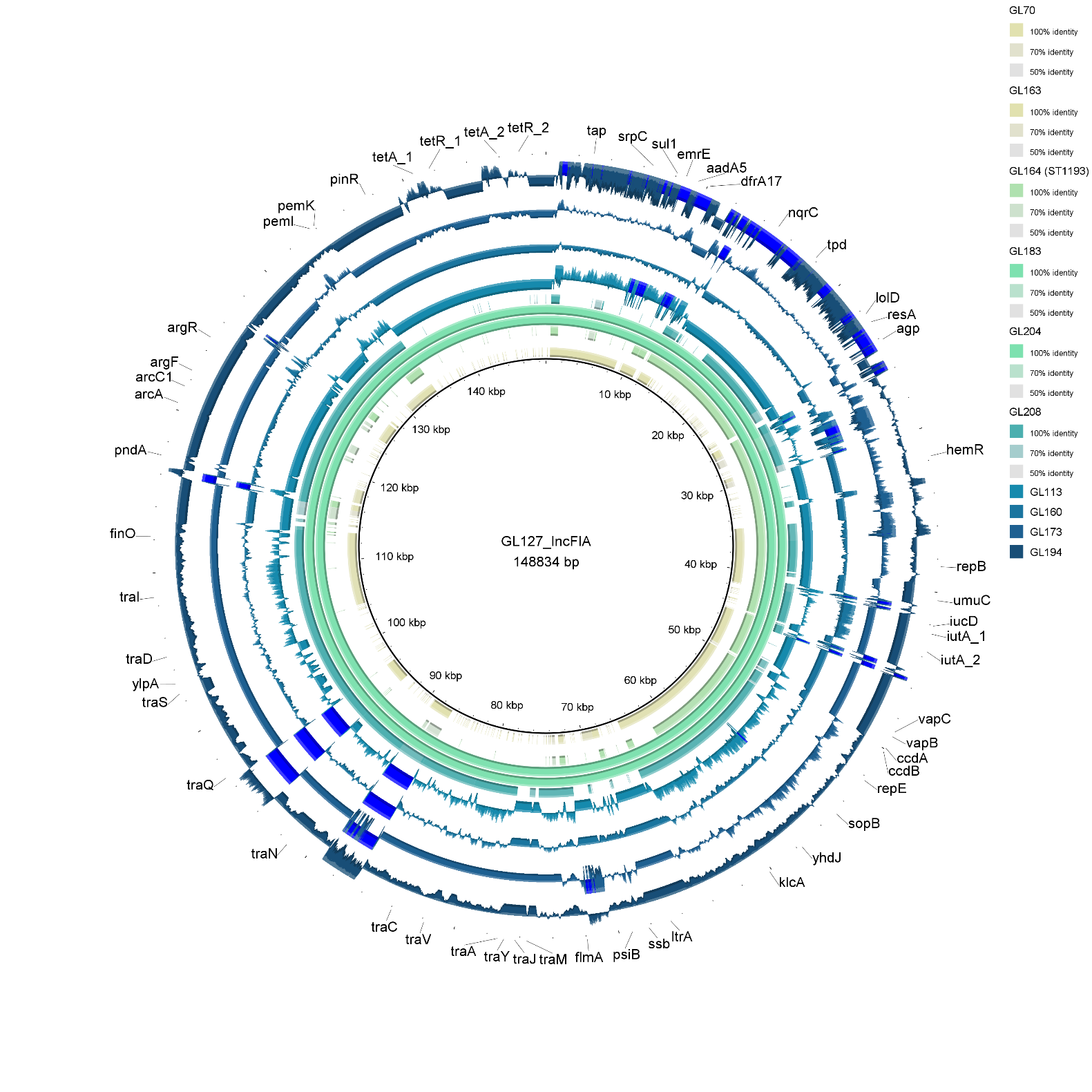

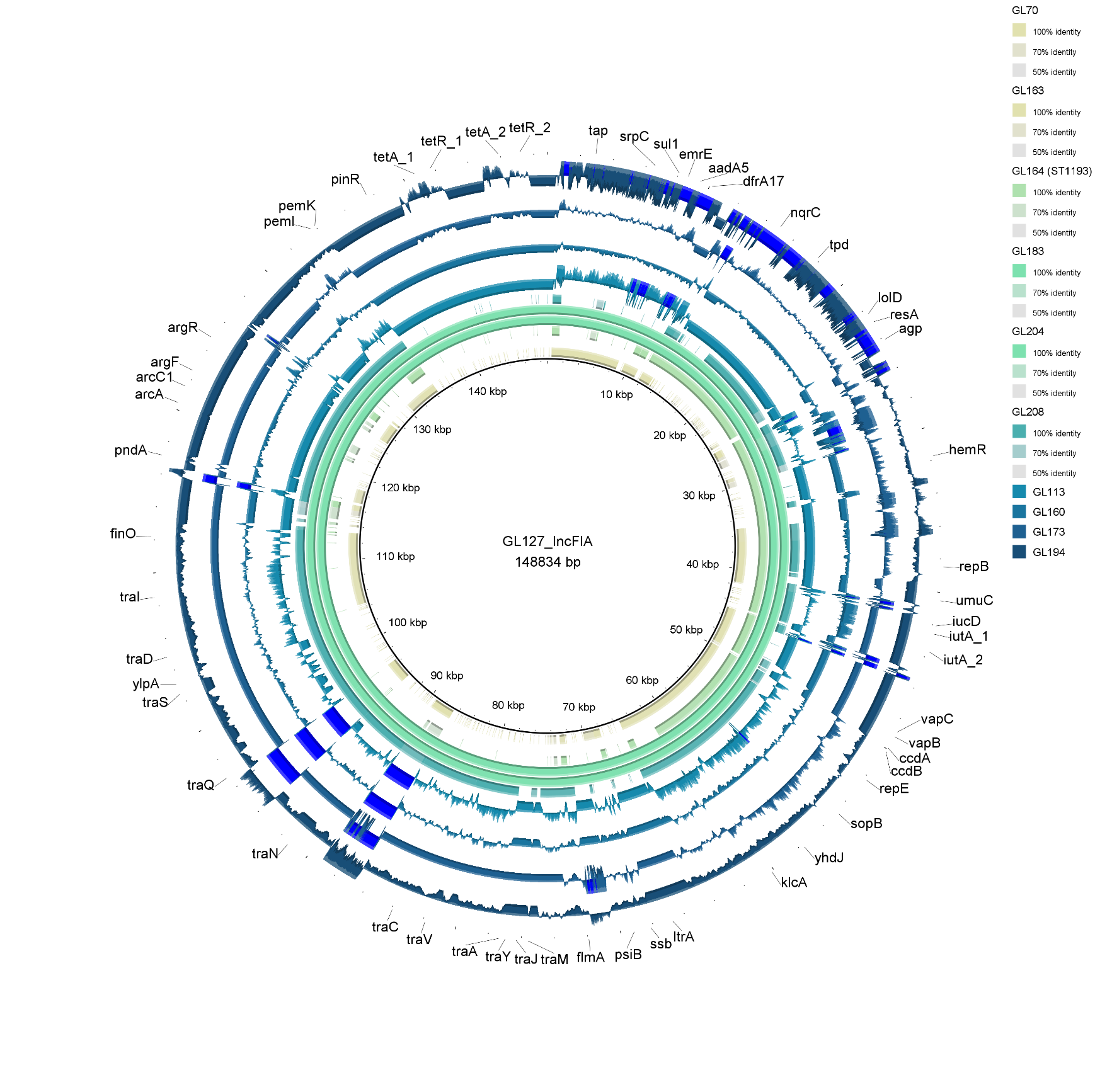


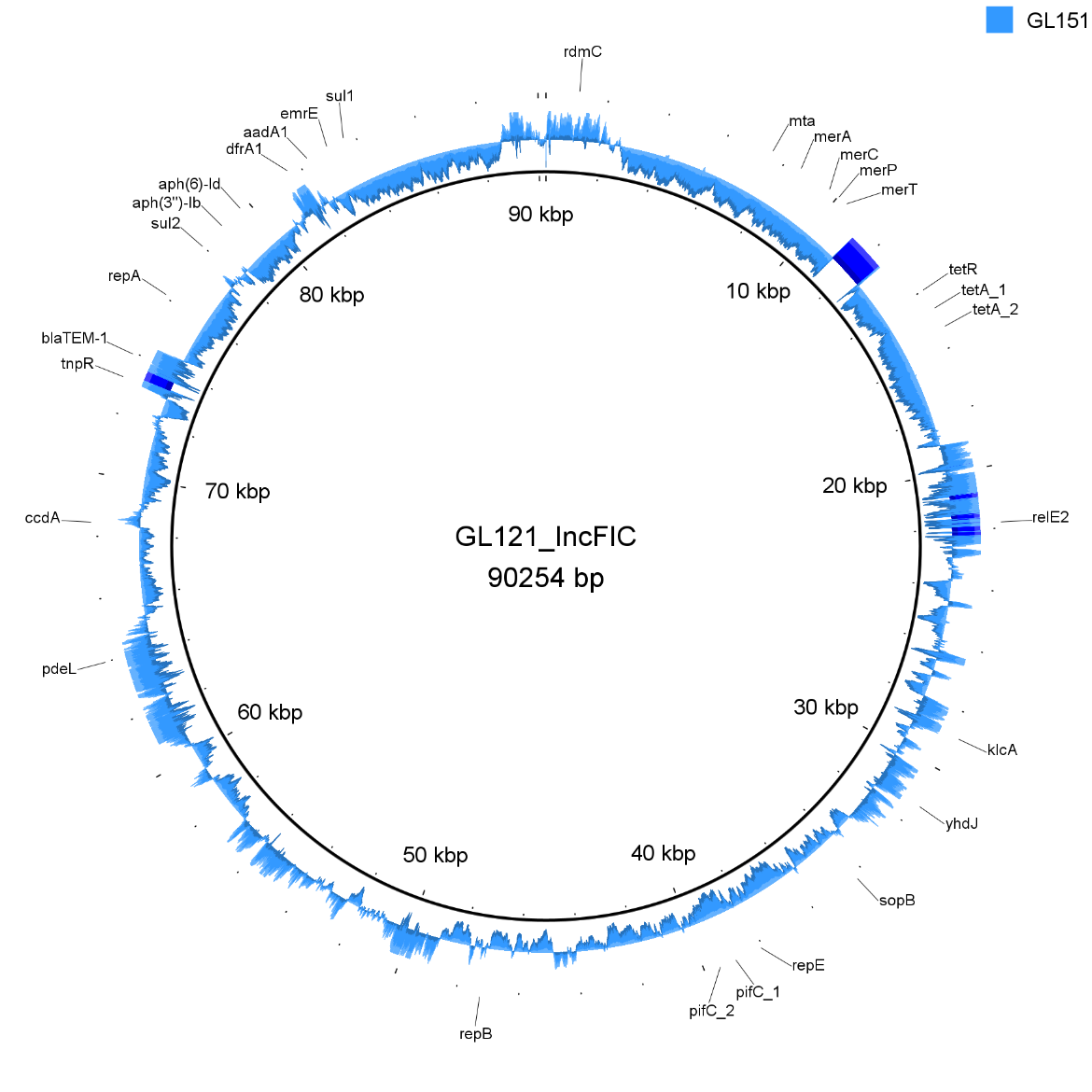

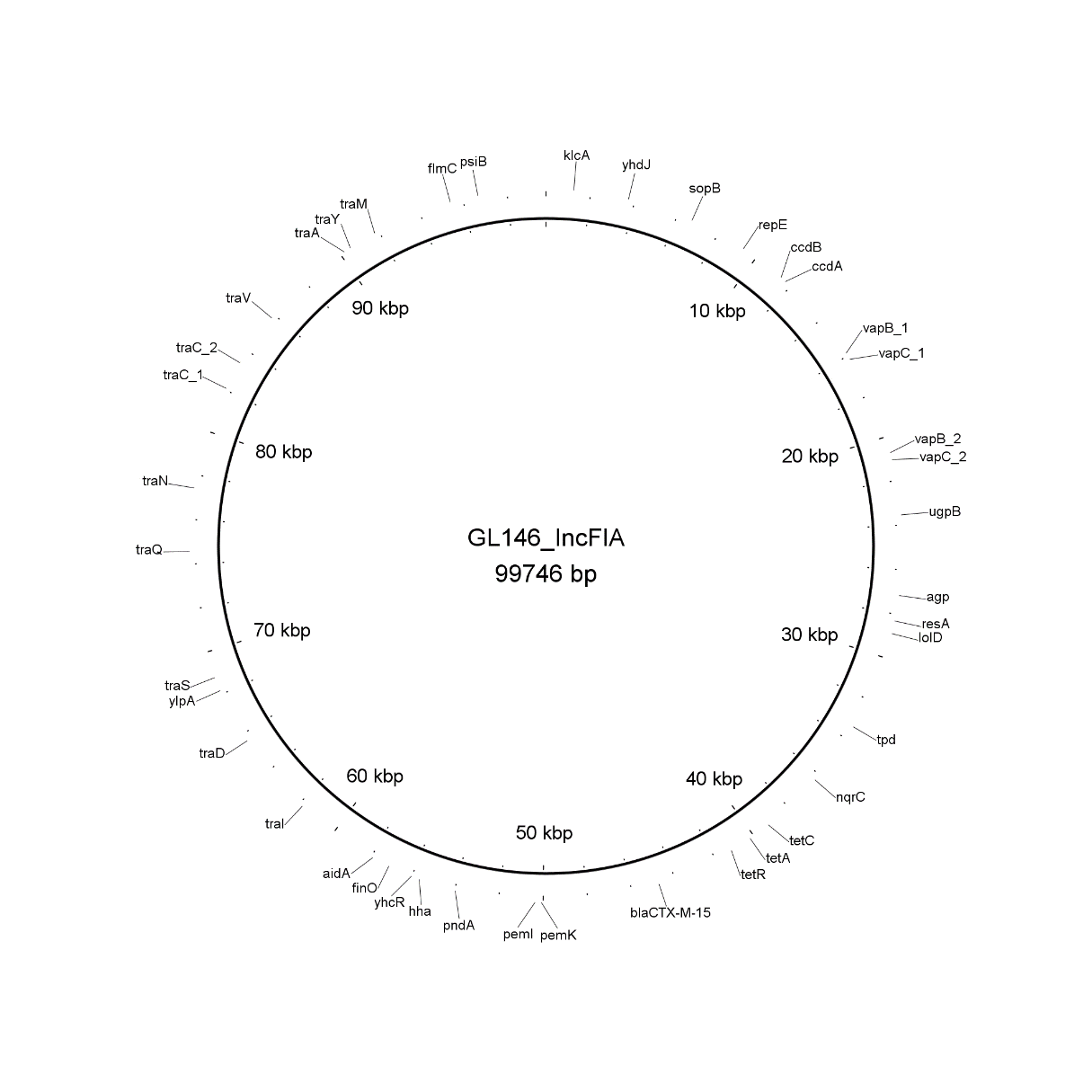


**ST3177**

**ST1633**

**Figure S1. Plasmids identified by long read sequencing with comparisons against isolates from the same ST harbouring a putative plasmid of the same type (except ST1193 which was added to ST131 comparison plot).** Plots are grouped by ST (labelled on top left corner) and each ring represents an isolate. Comparisons were made by either sequence similarity against contigs (when hybrid assembly was available; can be identified by isolates with identity range in legend) or coverage of short reads. Red/blue spikes in short read coverage indicate regions with one standard deviation from the mean coverage. Gene annotations of reference plasmid was added to the outer ring.
